# Cases Illustrating Plastic Surgery of the Face

**Published:** 1904-04

**Authors:** M. B. Hutchins

**Affiliations:** Atlanta, Ga., Dermatologist to Presbyterian Hospital


### CASES ILLUSTRATING PLASTIC SURGERY OF THE FACE.*

By M. B. HUTCHINS, M.D., Atlanta, Ga.,
Dermatologist to Presbyterian Hospital.

Case 1. Destruction of greater part of bony structure of nose and cartilaginous portion. Flat across bridge. Result of late hereditary syphilis cured three years before. Operation: Rhinoplasty. Chloroform. Assisted by Drs. Elkin and J. L. Campbell. The hole left in the site of the end of the nose was "trimmed out." Along each side a groove was made for edges of flap, and also a surface denuded at base of upper lip for the column of the new nose. With its attachment at the left upper part of the nose, a fan-shaped flap was dissected from the forehead, including all tissues to the periosteum. This flap was made larger than seemed needed, to allow for contraction: It was brought around to the right and down to the part to be repaired. The edges were attached in the grooves prepared for them and an imitation of the internostrillar column stitched in place. No effort was made to line the new nostrils, I hoping to be able to prevent cohesion of sur-

*Read at the January, 1904, meeting of Staff of Presbyterian Hospital.

faces by the use of rubber tubing. This was the cause of a great deal of trouble and was unsatisfactory. After the stitching was completed the entire face, from the mouth upward, was enveloped in exceedingly thick dressings. Five days later these were removed, and the new nose was found to have good circulation and union was far advanced. There was a slight inturn of the right edge of the flap, which I later tried to remedy by making a new groove in the cheek at the right, separating this side of the flap and reattaching it farther out, but the "roll" was never entirely obliterated. As soon as union was complete I severed the "handle of the fan"—the pedicle—and closed the wound left there. The forehead wound was allowed to heal by granulation.

So far I have described practically the average rhinoplasty. At the time of severance of the pedicle the skin over the site of the former bridge of the nose was split longitudinally to the nose, and the narrow part of the flap was fitted in this cleft and neatly stitched, instead of being cut away as in the classic operation. The patient was nineteen years old. Though it was a very unhandsome nose, she got married about six months after returning to her home.

Case 2. A woman of middle age. Epitheliomatous destruction of the right lower eyelid and some of the tissues below. Assisted by Dr. Grandy. The diseased area was carefully dissected away. A flap was then made by two parallel incisions the width of the orbit apart, downwards and a little outwards for about two inches—this flap being dissected from its attachments beneath. It was brought up and stitched closely to the thin remaining tissues below the eyeball, and the edges were united in their new position laterally. The result was excellent, though the substitute for an eyelid was imperfect. Later there was a slight recurrence of disease near the inner canthus, where the corner of the flap had sloughed. Caustic potash was used successfully.

Case 3. Rodent ulcer type of cancer on middle part of lower lip and chin. Operated July, 1899, assisted by Drs. Stockard and Sharp. Caustic potash and curette were first thoroughly used. The visibly affected area was then dissected away, leaving two little, separated ends of lower lip, the front of the chin being entirely uncovered, then skin down to larynx incised and loosened, and subcutaneous tissues, including a diseased gland, suprahyoid, removed. The submental incision was closed, lateral flaps of skin drawn as near as possible towards each other, and little remnants of lower lip united, strongly arching the upper lip. Perfect union below chin. As expected, none of stitching above of any use. October, 1899, the second operation, for purpose of forming a new lower lip, the chin-bone being then covered with fairly healthy granulations. The cheeks were split directly outward from the oral angles nearly to the masseter muscles. On each side, just below the jaw border, other incisions were made, so that the flaps retained the blood-supply of the facial arteries. These flaps were then brought together, forming a new lower lip and covering for the chin, the skin and mucous membrane being united to form the proper lip border, length of lower lip the same as that of the upper, and the shape of it almost as good as a natural lip. The chin part never served its purpose because of unpreventable infection and persistence of the disease, but the lip lasted over a year, and was very satisfactory to the patient. Later this case became rather celebrated because of the brilliant result of its treatment with the X-rays.

Case 4. Woman of sixty. Epithelioma had destroyed the right oral angle and progressed in a semi-circular manner into the tissues of the cheek. Under chloroform anesthesia, the visible disease was destroyed with caustic potash. After sufficient time had elapsed for the separation of all destroyed tissue, and the appearance of healthy granulating surfaces, chloroform was again administered (Drs. J. C. Johnson and W. B. Sharp assisted). The edges of the open area, which extended towards the border of the jaw and at the site of the facial artery, were pared, the curve of the wound above and below passing inward one-half inch or more on lips, but leaving a squared end of each lip remnant on the right. The opposite sides of the wound were stitched, beginning below, up to a point corresponding to the proper situation of the right oral angle. The flap, or square lip ends, were then turned downward or folded on themselves, the coapted raw surfaces so stitched as to meet the point where other stitches ended, thus forming a mouth of normal shape. The result was excellent.

*1007–1008 Century Building.*

